# Smokers’ Choroidal Changes

**DOI:** 10.1167/iovs.63.5.21

**Published:** 2022-05-18

**Authors:** Alessio Marino, Ciro Sannino, Nicola Rosa

**Affiliations:** 1 Eye Unit, University of Salerno, Baronissi, Italy. E-mail: cirosanninodr@gmail.com.

**Keywords:** OCT, CVI, choroidal vascular index, blooming effect

We read with great interest the article by Wei et al.^1^ concerning “Choroidal Structural Changes in Smokers Measured Using Choroidal Vascularity Index.”

We congratulate the authors for their very interesting study, but we would like to make some comments.

We are a bit concerned on the patients’ recruitment methods.

The authors compared smokers to nonsmoker patients, but it seems that the two groups were not correctly matched. In fact, not only was the non-smokers group younger than the smokers group, but the groups were not matched by axial length or at least refraction. This could have heavily influenced the results, as it is well known that patients with longer eyes have thinner choroids.

Moreover, the authors utilized the choroidal vascularity index (CVI), a parameter obtained converting a gray-scaled image into a new one, defined as “binary image,” allowing pixels to assume only two possible values, that is to say black or white, instead of a plethora of measures associated with different gray shades.

Even if CVI has been largely used in the international literature, in our opinion, it has an important limitation: it can be influenced by the so-called “blooming effect.”^2^^,^^3^ This is an artifact well known in the ultrasonography field^4^^,^^5^ that makes it difficult to obtain reliable measurements, mainly if they are very small, as in the case of the ocular and orbital ones.^6^^,^^7^ This effect is related to the signal amplification (i.e. when high signal amplification is used, the image will appear brighter and the number of white pixels will be greater); indeed, the exact opposite phenomenon will happen when using low signal amplification.^8^^,^^9^

This artifact seems to be present also in case of optical coherence tomography (OCT): this is why we are afraid that this effect could also influence the binarization utilized in the CVI evaluation, increasing the low reflective areas (considered to be associated with luminal ones) when the amplification is low, whereas reducing them when the amplification is higher.

For all these reasons, in our opinion, the flaws presented in this study cannot allow a definitive and reliable conclusion.

## References

[1] Wei X, Kumar S, Ding J, Khandelwal N, Agarwal M, Agrawal R. Choroidal Structural Changes in Smokers Measured Using Choroidal Vascularity Index. *Invest Ophthalmol Vis Sci*. 2019; 60(5): 1316–1320. 30943279 10.1167/iovs.18-25764

[2] Rosa N, Vitiello L, De Bernardo M. Optic nerve sheath diameter measurement in hypoxic ischaemic brain injury after cardiac arrest. *Resuscitation*. 2019; 138: 310–311. 30878376 10.1016/j.resuscitation.2019.01.043

[3] De Bernardo M, Rosa N. Optic nerve sheath diameter measurement in patients with idiopathic normal-pressure hydrocephalus. *Eur J Neurol*. 2018; 25(2): e24 29356262 10.1111/ene.13530

[4] Rosa N, De Bernardo M. Ultrasound assessment of optic nerve sheath diameter in healthy volunteers. *J Crit Care*. 2017; 40: 279. 28372887 10.1016/j.jcrc.2017.03.018

[5] De Bernardo M, Vitiello L, Rosa N. Ultrasound optic nerve sheath diameter evaluation in patients undergoing robot-assisted laparoscopic pelvic surgery. *J Robot Surg*. 2019; 13: 709–710. 31028530 10.1007/s11701-019-00966-7

[6] De Bernardo M, Vitiello L, Rosa N. Optic nerve ultrasound measurement in multiple sclerosis. *Acta Neurol Scand*. 2019; 139(4): 399–400. 30723917 10.1111/ane.13072

[7] De Bernardo M, Rosa N. Comment on ‘Invasive and noninvasive means of measuring intracranial pressure: A review’. *Physiol Meas*. 2018; 39(5): 058001. 29767627 10.1088/1361-6579/aac540

[8] Iaconetta G, De Bernardo M, Rosa N. Coronal Axis Measurement of the Optic Nerve Sheath Diameter. *J Ultrasound Med*. 2017; 36(5): 1073. 10.1002/jum.14198 28390163

[9] De Bernardo M, Marotta G, Rosa N. Sonography of the Optic Nerve Sheath Diameter. *J Ultrasound Med*. 2018; 37(7): 1845. 29148071 10.1002/jum.14486

